# Supplementary material for: Targets for Renal Carcinoma Growth Control Identified by Screening FOXD1 Cell Proliferation Pathways
Source: Cancers (Basel). 2022 Aug 16;14(16):3958. doi: 10.3390/cancers14163958 (PMC9406217; doi:10.3390/cancers14163958)
Supplement: Supplementary file 1 [file cancers-14-03958-s001.zip › cancers-1798341-supplementary.pdf]

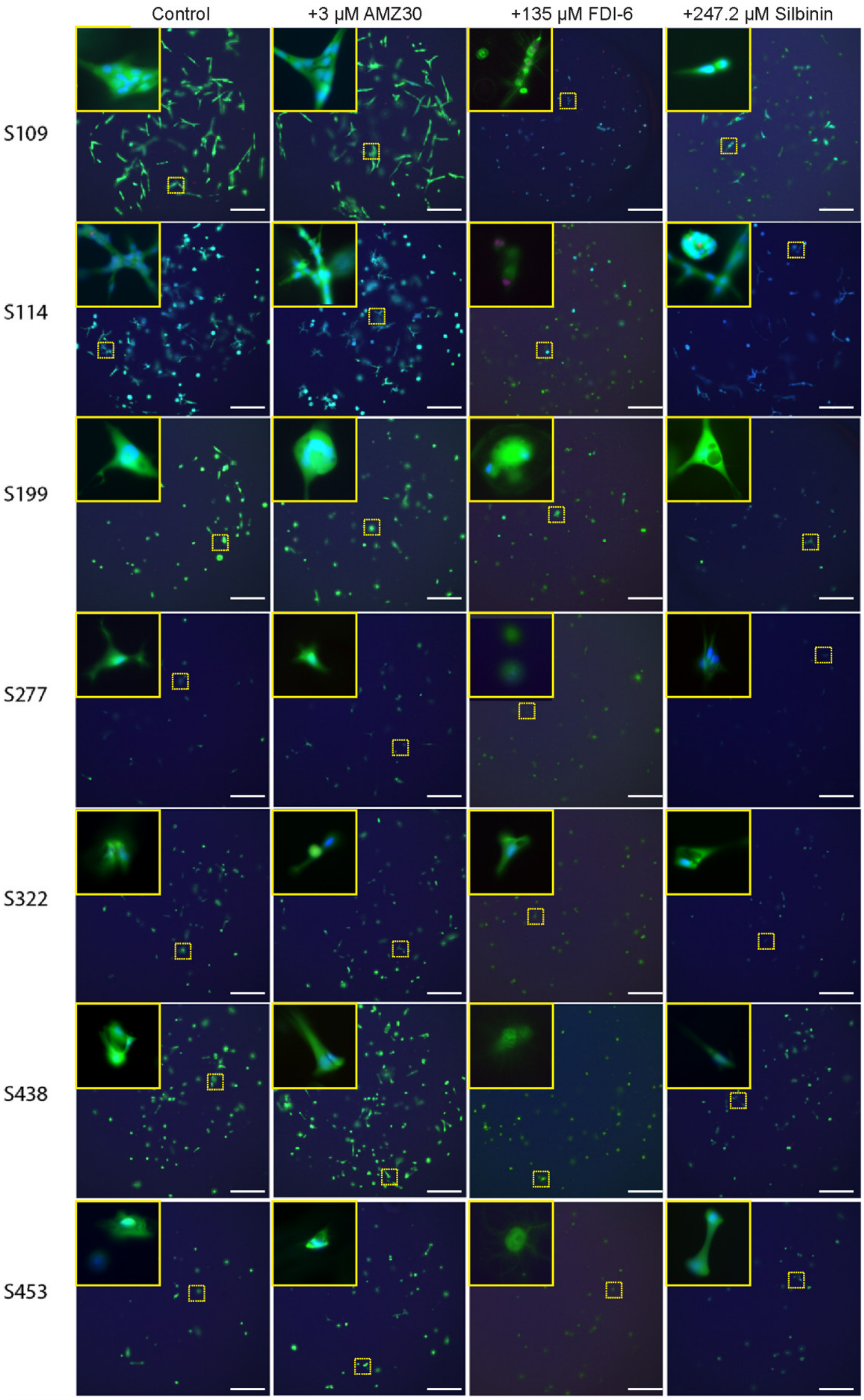

Figure S1: Compound treatment of patient tumor avatars. Scalebar = 500 microns.

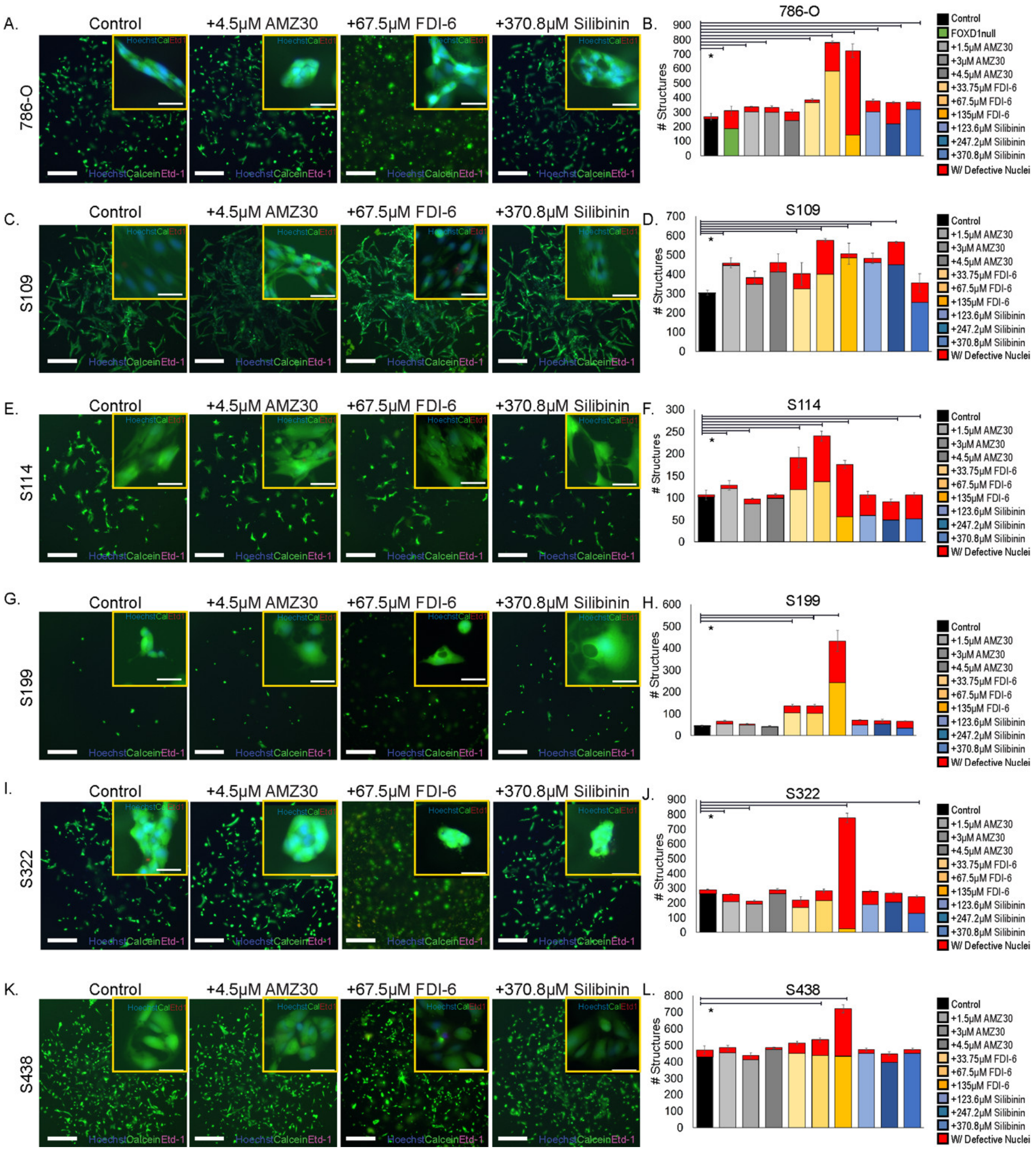

Figure S2: Compound treatment of primary 3D cultures structure analysis. (A-L) Treatments of primary 3D tumor cultures with compounds (AMZ30, Silibinin, FDI-6) were titrated and analyzed for number of structures formed. Scalebar = 500µm. Inlay images are representative structures. Scalebar = 20µm.

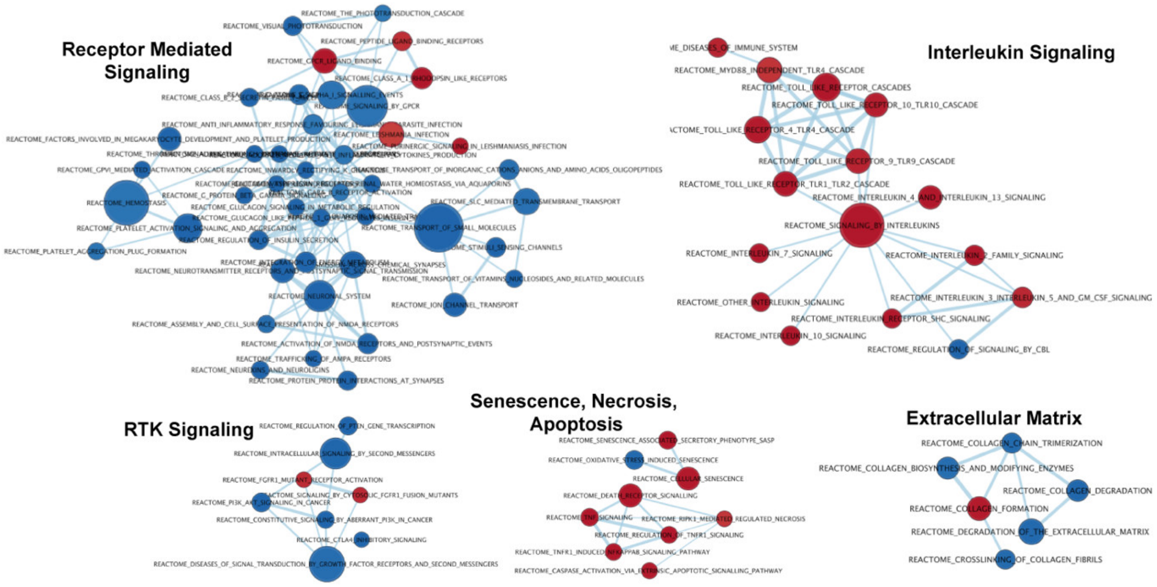

Figure S3: GSEA Pathway analysis identifies actionable pathways. Red nodes indicate REACTOME pathways upregulated in 786-O^{FOXD1null} while blue nodes indicate pathways downregulated in 786-O^{FOXD1null}.

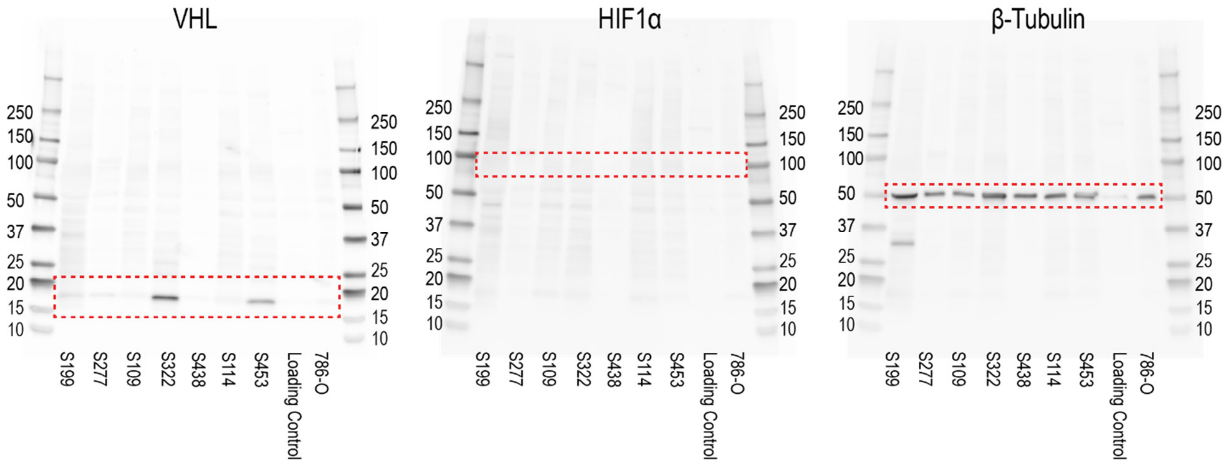

Figure S4: Uncropped Western Blot Figures. Unedited blots used in Figure 8A. Dotted-red line indicates region analyzed for protein abundance against designated target. Contrast enhanced in main figure for presentation.
