# Supplementary material for: Targets for Renal Carcinoma Growth Control Identified by Screening FOXD1 Cell Proliferation Pathways
Source: Cancers (Basel). 2022 Aug 16;14(16):3958. doi: 10.3390/cancers14163958 (PMC9406217; doi:10.3390/cancers14163958)
Supplement: Supplementary file 1 [file cancers-14-03958-s001.zip › Table S2.pdf]

Table S2: Compound titrations used in study.

Compound	Target	Reported Concentration (& Source of report)	Reported Assay
AMZ30	PME1	IC50 = 0.6μM (Calbiochem)	Human cell protein lysates and HEK293T methylated PP2A measurements.
EED226	SUZ12	IC50 = 53.5nM (Selleckchem)	Human G401 Cells *in vitro* enzymatic assay.
CINPA1	CXADR	IC50 = 70nM (EMD Millipore)	Human primary hepatocytes CAR-mediated transcription analysis.
Galectin-9 mAb	LGALS9	1 IU = 1 μg/ml (Sigma-Aldrich)	Human CD14+ PBMCs expression of CD40, CD54, and CD80.
Recombinant Semaphorin 3C	SEMA3 C	Reported = 10 μg/ml (R&D Systems)	Mouse SVEC-4-10 Cells binding to fibronectin.
Recombinant CXCL5	CXCL5	ED50 = 3 – 15ng/ml (R&D Systems)	Chemoattraction of CXCR2+ mouse BaF3 cells .
Recombinant ANGPTL4	ANGPT L4	IC50 = 0.05-2μg/ml (R&D Systems)	Chemical assay inhibition of lipoprotein lipase activity.
Recombinant SHH	HHIP	ED50 = 0.1 – 0.42 μg/ml (R&D Systems)	Mouse C3H10T1/2 alkaline phosphatase assay.
Recombinant CTRP1	C1QTN F1	ED50 = 0.6- 3.62 μg/ml (R&D Systems)	Functional ELISA protein bindin.g to recombinant BAI3
Recombinant Netrin	UNC5B	ED50 = 0.5 – 2.0 μg/ml (R&D Systems)	Rat RT4-D6P2T proliferation assay.
WAY1701523	MMP1 3	IC50 = 17nM (Tocris,[25])	Alkaline phosphatase activity of AP- PAR1 rat cardiac fibroblasts.
C5OH	S100P	IC50 = 100nM ([43])	NF-κB activity of human BxPC-3 cells expressing NF-κB-luc.
CPI-637	EP300	IC50 = 0.05μM (Selleckchem)	TR-FRET Assay inhibition of CBP/EP300 in human AMO-1 cells.
FDI-6	FOXM1	IC50 = 22.5μM (Sigma-Aldrich)	Proliferation inhibition of human MCF-7 cells.
APE-1 Inhibitor III	APEX1	IC50 = 600nM - 12μM (EMD Millipore)	Alkaline phosphatase activity inhibition of APE1 in human HEK293T and HeLa cells.
Benezyl Isocyanate	AGPS	IC50 = 2 - 5μM ([33])	Inhibition of proliferation of human MDA-MB-231 cells.
Silibinin	TMEM 167A	IC50 = 30.9μM	Proliferation of human fibroblasts, E.coli ATP synthase activity, and gene expression of prostate cancer.
Recombinant LTBP1	LTBP1	IC50 = 2μg/ml ([48])	Immunolocalization with extracted fibrillin-1.
